# Using an Automatic Resistivity Profiler Soil Sensor On-The-Go in Precision Viticulture

**DOI:** 10.3390/s130101121

**Published:** 2013-01-16

**Authors:** Roberta Rossi, Alessio Pollice, Maria-Paz Diago, Manuel Oliveira, Borja Millan, Giovanni Bitella, Mariana Amato, Javier Tardaguila

**Affiliations:** 1 Sistemi Colturali degli Ambienti Caldo–Aridi (SCA), Agricultural Research Council (CRA), Via Celso Ulpiani, 5, 70125 Bari, Italy; E-Mail: roberta.rossi@entecra.it; 2 Department of Economics and Mathematics, University of Bari, 70124 Bari, Italy; E-Mail: alessio.pollice@uniba.it; 3 Instituto de Ciencias de la Vid y del Vino (University of La Rioja, CSIC, Gobierno de La Rioja) 26006 Logroño, Spain; E-Mails: mpaz.diago.santamaria@gmail.com (M.-P.D.); borja.millanp@unirioja.es (B.M.); 4 CITAB, Department of Agronomy, UTAD, 5001-911 Vila Real, Portugal; E-Mail: mto@utad.pt; 5 So. In. GStrutture & Ambiente, 57121 Livorno, Italy; E-Mail: g.bitella@soing.eu; 6 School of Agriculture, Forest and Environmemtal Sciences, University of Basilicata, Viale dell'Ateneo Lucano, 10, 85100 Potenza, Italy; E-Mail: marinria@gmail.com

**Keywords:** soil spatial variability, electrical resistivity, vineyard variability

## Abstract

Spatial information on vineyard soil properties can be useful in precision viticulture. In this paper a combination of high resolution soil spatial information of soil electrical resistivity (ER) and ancillary topographic attributes, such as elevation and slope, were integrated to assess the spatial variability patterns of vegetative growth and yield of a commercial vineyard (*Vitis vinifera* L. cv. Tempranillo) located in the wine-producing region of La Rioja, Spain. High resolution continuous geoelectrical mapping was accomplished by an Automatic Resistivity Profiler (ARP) on-the-go sensor with an on-board GPS system; rolling electrodes enabled ER to be measured for a depth of investigation approximately up to 0.5, 1 and 2 m. Regression analysis and cluster analysis algorithm were used to jointly process soil resistivity data, landscape attributes and grapevine variables. ER showed a structured variability that matched well with trunk circumference spatial pattern and yield. Based on resistivity and a simple terrain attribute uniform management units were delineated. Once a spatial relationship to target variables is found, the integration of point measurement with continuous soil resistivity mapping is a useful technique to identify within-plots areas of vineyard with similar status.

## 1. Introduction

The quest for tools, instrumentation and sensors to assist management decisions in agriculture is increasingly complex, given the availability of sophisticated tools which needs to be matched by appropriate testing and validation. Wine grapes are a high-value crop and investments that improve production efficiency and enhance quality can be well rewarded [1]. In this context precision viticulture, a technological driven concept, that exploits the knowledge of within-field spatial variability to design site-specific optimal management strategies [2,3], holds great promises. Its applicability, though, depends on both availability and accuracy of spatially referenced information [4].

Soil is a key factor in viticulture and wine composition is influenced by the soil-plant interaction [5]. Soil information can be used prior to vineyard plantation reduce future crop variability within a vineyard area [6]. In this regard, soil spatial variability in a vineyard was showed to be related to vegetative growth, yield components and grape composition [7–9] and to influence the spatial variation of wine sensory attributes [10].

Soil variability of the vineyard is traditionally investigated by destructive sampling on a limited number of sites [7]. Destructive sampling though, besides being labor intensive and time consuming, can be misleading if sampling distances are chosen without any prior knowledge of inherent soil spatial variability [11]. To produce reliable maps of soil variability by any interpolation method, a large number of samples must be available and sampling distances must be related to the spatial structure of the target variable. In this context the use of ancillary data, such as topographic attributes and proximal/remote sensing data, can help revealing the scale of variation of underline soil properties to optimize the choice of the sampling distances.

The great potential of geophysical measurements for characterizing soil spatial variability has been widely recognized in soil science [12,13]. Over the last decade, several researches provided a comprehensive insight on the use of electrical resistivity (or its inverse, electrical conductivity) as a proxy of soil physical and chemical properties. These techniques were used to monitor changes in dynamic soil properties [14,15] to discern the effects of management on soil structure [16,17] and when tested across different soils at different time of the year they have shown consistent correlations with permanent soil properties [18].

The development of continuous resistivity/conductivity sensors gave great impulse to the understanding of landscape-scale soil processes and they have been widely used for delineating uniform management zones together with terrain attributes and yield data [19–21]. Continuous resistivity/conductivity sensors currently available on the market can be divided into two broad categories: the non-invasive electromagnetic induction systems (EMI sensors) and the invasive electrode based direct current (DC) resistivity sensors. Each sensor has its operational advantages and drawbacks [22]. EMI sensors have the advantage of not requiring direct contact with the surface, while DC sensors need a solid contact, thus dry conditions or frozen surfaces prevent their use. On the other hand, EMI survey requires repeated calibrations prior to mapping and during the day because air temperature and humidity affect the measurements; DC sensors do not require field calibration and are not prone to field drift [23]. A specific limititation on the use of EMI instruments in viticulture might be their great sensitivity to electrical power fields such as power lines and running engines. As documented by [24] one of the drawbacks of this technique in viticulture is the confounding effects that can be generated by vineyard trellising, dripper irrigation pipes and guide wires. The authors recommend extreme care taken by the operator in keeping the antenna/sensor unit always in the mid-row of any transect, and advise to avoid this technique in vineyards containing still posts with less than 3 m row spacing. Moreover any changes in row spacing or trellising within the vineyard must be taken into account in data post-processing.

In the context of DC sensors, an automatic on-the-go recording resistivity meter, the Automatic Resistivity Profiling (ARP^©^) device has been developed specifically for agriculture and permits fast and extensive soil mapping through apparent electrical resistivity measurements. First results in viticulture [25] demonstrated that ARP can overcome the major limits of EMI related to interference with metallic wires. The ARP multi-electrode continuous profiler represents a technological advancement since it permits fast and extensive soil mapping through apparent electrical resistivity measurements. The equipment is towed along the field to collect data simultaneously at different depths corresponding to the distance between injecting and receiving wheels, and the spatial information is used for positioning measurements and computing of a Digital Elevation Model (DEM) providing topographic attributes such as slope and position that facilitate the interpretation of resistivity variation and the definition of management zones [26].

However, there are a number of open issues before soil sensor can be routinely used in world viticulture to study the vineyard spatial variability. The main objective of this work was to study the potential use of a direct current (DC) continuous resistivity profiling on-the-go sensor in precision viticulture and to explore its relationship with the vine trunk circumference and crop yield.

## 2. Materials and Methods

### 2.1. Experimental Layout

The study was conducted in a 3.5 hadry-farmed cv. Tempranillo (*Vitis vinifera* L.) commercial vineyard, located in Logroño (42°28′44″N, 2°29′35″W, 493 m), in the wine-producing region of La Rioja, Spain. Grapevines were grafted on 110 rootstock and planted in 1990, following a 2.8 m × 1.2 m (inter- and intra-row) spacing, and trained to a vertically shoot-positioned (VSP), spur-pruned cordon with a bud load of 11 nodes per vine. Standard vineyard management operations conducted in the Rioja Appellation District were followed. The vineyard area was a 7% linear concave slope facing South-east, and its soil was an *Inceptisol* with three sub-divisions: *Petrocalciccal cixerept*, *Typical cixerept*, and *Calcic haploxerept* [27]. The maximum difference in soil elevation in the vineyard plot was about 5 m.

A regular grid of 65 experimental blocks was defined using a Leica Zeno 10 GPS (Heerbrug, St. Gallen, Switzerland), with real time kinematic correction, working at <30 cm precision, and each experimental block comprised three vines chosen in two adjacent rows.

### 2.2. ARP Sensor

**Figure 1.**
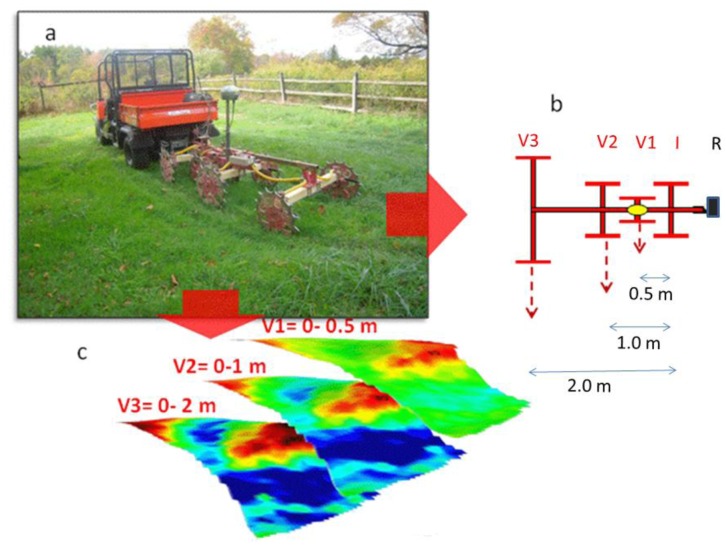
(**a**) Picture of the DC recording resistivity meter (ARP^©^, Automatic Resistivity Profiling. GEOCARTA, Paris, France) towed by a ground vehicle; (**b**) schematic representation of the multiple system: R: resistivity meter; I: injection wheel; V1, V2 and V3: receiving wheels (**c**) maps of resistivity distribution at the three consecutive exploration depth (V1 = 0−0.5 m; V2 = 0−1.0 m; V3 = 0−2.0 m).

The sensor used for continuous resistivity measurement was an automatic on-the-go DC recording resistivity meter (ARP^©^, Automatic Resistivity Profiling. Geocarta, Paris, France) (Figure 1(a)) which consisted of the following settings [23]:

- A multipole made of four pairs of rolling electrodes arranged in V-shaped configuration specifically designed to optimize the acquired signal quality. One pair of rolling electrodes is used to inject current in the soil and the other three pairs function as receiving electrodes and areset at a distance of 0.5, 1 and 2 m from injecting electrode wheels (Figure 1(b)), in order to measure apparent soil electrical resistivity (ER) for a depth of investigation up to 0.5, 1 and 2 m respectively. Resulting measurements therefore respectively refer to the 0–0.5 m depth layer (V1), the 0–1.0 m depth layer (V2), and the 0–2.0 m depth layer (V3) (Figure 1(c)).
- A resistivimeter, designed for optimized synchronous measurement of three channels with a quick time response (44 ms) and a high tolerance to contact resistance that allow high-speed measurements.
- A Doppler radar to trigger the ER measurements at 20 cm intervals along each transect. A computer, to display and store the values of apparent electrical resistivity, which is equipped with a GIS software Geocarta Office (Geocarta, Paris, France), allowing data to be processed in real time.
- An absolute positioning system (DGPS) for real-time data referencing.

### 2.3. Soil Resistivity Measurements

Electrical profiling was conducted on 25 November 2011 in a 3.5 ha Tempranillo vineyard using the ARP towed by a ground vehicle through the vineyard along parallel transects at the distance of approximately 5.60 m between transects. This distance corresponded to an ER transect every two vine rows. Transects covered a length of 7,887 m. A total number of 115,510 measurements were taken. The entire area was surveyed in about 43 minutes, at an average speed of 9.96 km/h. DGPS data were used to produce a Digital Elevation Model (DEM).

### 2.4. Grapevine Vigour and Crop Yield Measurements

**Figure 2.**
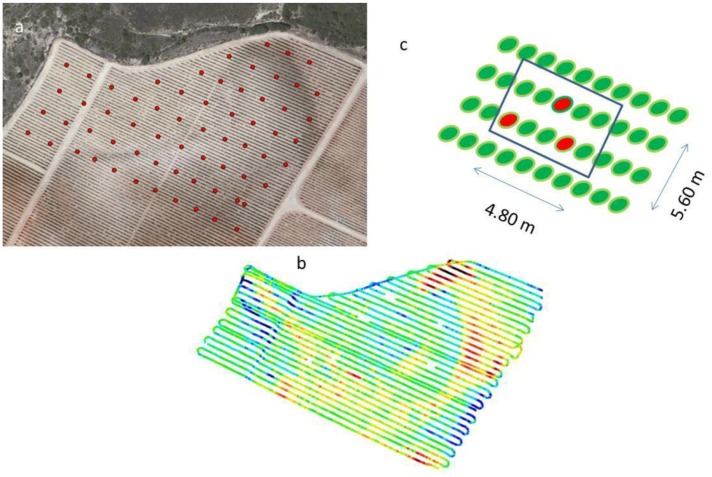
Field measurement locations (**a**) aerial photograph of the field with the position of plant measurement sites (red dots: center of each plant measurement block). (**b**) electrical resistivity transects with average ER values for layer 1 (0–50 cm depth). (**c**) scheme of a typical plant block with the three vines (in red) used for measurements.

The 65 experimental 3-vine blocks of the sampling grid (Figure 2(a)) were measured prior to harvest (September 2008) for trunk girth above the grafting insertion. The three vines were chosen in two adjacent rows as indicated in Figure 2(c). At harvest (8 October 2008), the yield of each vine was weighted in the field and the average yield value of the three vines per experimental block was calculated.

### 2.5. Statistical, Geostatistical Analysis and Mapping

Summary statistics of ER data measured in the three layers (0–0.5 m; 0–1 m; 0–2 m) were computed and correlation between ER strata was calculated. To display ER spatial pattern outlying observation were removed and data roughness was reduced using a 2D median filtering algorithm (GEOCARTA Office; GEOCARTA SA, Paris, France). Smoothing of the data surface is obtained considering the median of values observed over an area centered at each data point:

(1) y(m,n)=median[x(i,j),(i,j)\inw]

where *x*(*i*,*i*) are the observed data, *y*(*m*,*n*) are the filtered data, *m* and *n* are the coordinates of *y* and *w* represents an area centered around the location (*m*,*n*).

Although median filtering may round-off corners, it was chosen due to its capability to preserve edges while removing noise of many types [28], and to neither shift boundaries nor reduce contrast across steps, since no new values are introduced in the smoothing process, as would happen with mean filtering.

After filtering data were interpolated by a cubic spline interpolation. Elevation data were used for the computation of a Digital Elevation Model using Golden Software Surfer v10. The terrain attribute of slope was calculated using a terrain-modelling algorithm in Quantum GIS (QGIS) 1.8.0.

Plant variables and ER were measured at a different sampling scale as ER has a much finer resolution. In order to measure the association between these misaligned data, a smoothing procedure was used to reduce the roughness of ER data. A 2D median filtering process was performed on ER data using 12.5 × 12.5 m neighborhood matrix. Once the ER spatial resolution was reduced to that of the observed vines, several scouting data analyses were performed in the ‘R software environment for statistics’ (www.r-project.org).

Graphs allowed identifying outlying locations, summarizing distributional features and highlighting the outstanding behavior of groups of observations. Maps and contour plots were obtained as follows: first a 40 × 40 points orthogonal rectangular grid was overlaid to the study area, then values at grid points were calculated by linear interpolation within triangles formed by the three adjacent vines. Such interpolation corresponds to the default settings of function *interp* in the R library *akima*. Empirical semivariograms of the girth of trunk, yield and electrical resistivity were estimated by the method of moments for 13 distance classes, specifying a maximum distance of 150 m by function *variog* in the R library *geoR*. Theoretical variograms where then manually overlaid using function lines, variomodel in the same library. Causal dependence of vegetative development and yield on terrain attributes and electrical resistivity was assessed by linear and additive models. At an initial stage, first linear models were fit by OLS (Ordinary Least Squares, R function *lm*), checking the significance of each predictor variable (*t* test) and the overall model significance (*F* test). Residual analysis was performed throughout to assess the assumptions of Gaussianity and independence, including maps, contour plots and variograms of residuals. Even if in both cases there was no evidence of residual spatial variability, a spatially correlated random term was included in the model estimated by GLS (Generalized Least Squares, function *gls* in the R library *nlme*). Several specifications of the spatial trend and covariance model lead to estimated correlation structures characterized by meaningless values of the range parameter far below the minimum distance between two vines.

Alternatively, whenever evidence of a nonlinear spatial pattern was visible in the map of residuals, a smooth function of the observation coordinates was added to the linear model, namely an anisotropic bivariate smooth represented using tensor product splines was considered. Models including such a nonlinear trend surface belong to the Generalized Additive Models (GAM) class and are fitted by penalized likelihood maximization with function *gam* in the R library *mgcv*. The degree of smoothness of the trend surface was estimated as part of fitting by minimizing a Generalized Cross Validation (GCV) criterion.

### 2.6. Delineation of Uniform Management Zones

Based on the regression analysis results, uniform management zones coupling resistivity and slope were delineated. Fuzzy cluster analysis was carried out using an unsupervised continuous classification procedure using a fuzzy *c*-means implemented by Management Zone Analyst (MZA) software [29]. Significance differences in trunk girth average values were tested by ANOVA and Tukey HSD tests.

## 3. Results and Discussion

### 3.1. Gross Variation

**Figure 3.**
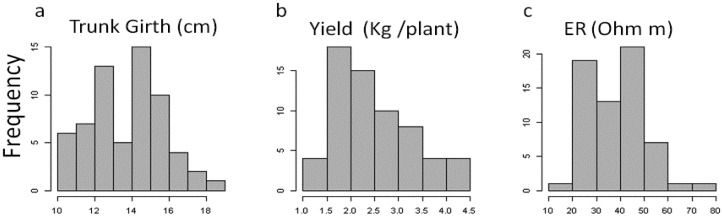
(**a**) Frequency distribution of trunk girth, (**b**) yield and (**c**) soil electrical resistivity (ER). All plant and ER data corresponded to 65 experimental sampled blocks of regular grid.

Figure 3 shows the frequency distribution of plant variables and of the subset of ER values at the corresponding locations. Plant attributes showed a marked variability, with values of trunk circumference ranging from 10.67 to 18.50 cm, and yield ranging from 1.22 to 4.27 kg per plant. ER and trunk circumference showed a bimodal distribution, while a unimodal right skewed frequency distribution was observed for the yield per vine.

Correlations were found between ER measured in the three layers and plant variables but the highest correlations were found with first layer (0–0.5 m). Topsoil ER was negatively correlated to trunk circumference (*r* = −0.40, *P* < 0.05) and yield (*r* = −0.40, *P* < 0.05).

### 3.2. Spatial Variability and Geostatistical Analysis

**Figure 4.**
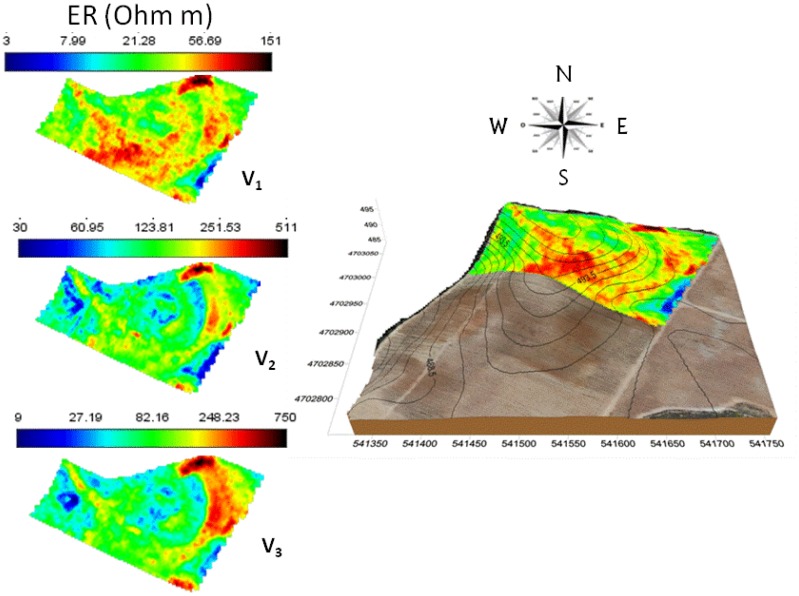
Interpolated maps (**left**) of ER measured by the ARP system in the three soil layers (V_1_ = 0–0.5, V_2_ = 0–1 and V_3_ = 0–2 m depth). Interpolated map (**right**) of the first layer (0–0.5 m) overlaid on the digital elevation model (DEM) of the experimental vineyard.

After outliers were removed and data roughness was reduced, ER values in the three layers were interpolated (Figure 4). The lowest values were found in the 0–0.5 m layer and the highest in the 0–2 m. ER values measured at the same location in the three ARP layers (V1, V2 and V3) were highly correlated between them (r_V1,V2_ = 0.81, r_V2,V3_ = 0.88, *P* < 0.001) and displayed a similar spatial pattern.

The spatial pattern of ER partially followed topographical gradients: a narrow resistive band (red-yellow areas) could be delineated at the top of the hill and along the SE facing ridge whereas high resistivity values (>90 Ohm·m) were measured along the southern border of the field, again following the morphological gradient over a shoulder slope. Peaks of resistivity (red-black areas, with ER values progressively increasing with depth) were measured in two areas situated in the NE and SE vineyard borders. In these zones, a large number of rock fragments on the surface were observed. Lower resistivity was measured in the lower part of the field, where finer-textured soils and high organic matter content was found [7]. These trends reflect the general expected relationship between electrical conductivity and landscape attributes. Jaynes *et al.* [30] found low conductivity near hill and ridges were soils are well-drained and coarser-textured and high values in lower portion of the landscape enriched by lower size sediments and high levels of organic matter content.

**Figure 5.**
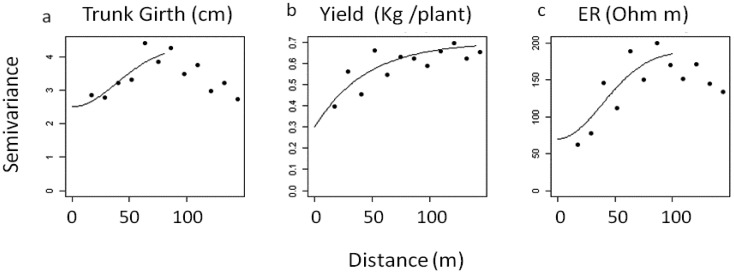
Empirical variograms of (**a**) trunk girth, (**b**) yield and (**c**) soil electrical resistivity (ER) overlaid by theoretical variogram models.

The variograms for ER and plant variables showed that both trunk girth and yield displayed a structured spatial variability pattern (Figure 5) consistent with ER, and similar ranges (between 45 and 55 m).

**Figure 6.**
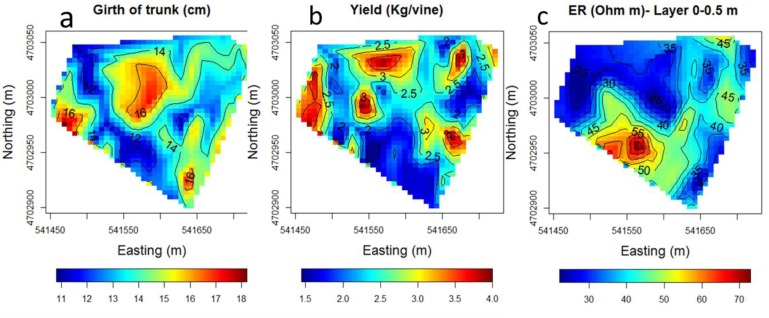
Interpolated maps of (**a**) trunk girth (cm), (**b**) yield (Kg/vine) and (**c**) top-layer (0–0.5 m) soil electrical resistivity (Ohm·m).

The semivariance of both trunk circumference and ER decreased rapidly beyond that range thus indicating the presence of clustering. The interpolated maps of trunk girth, crop yield and soil ER are depicted in Figure 6.

**Figure 7.**
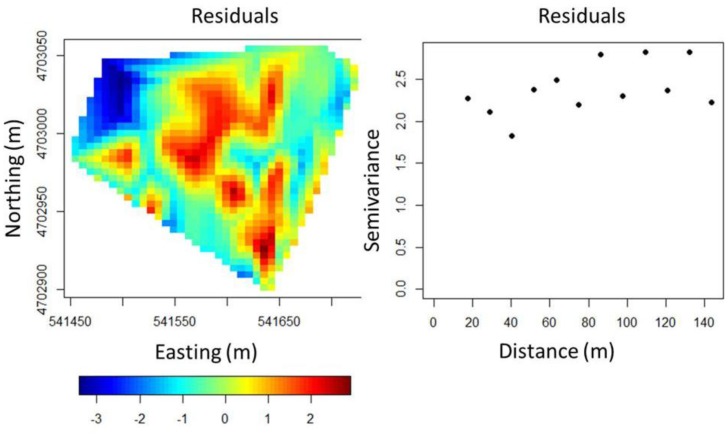
Map of residuals (**left**) and empirical semivariogram of residuals (**right**) of the linear multiple regression model for trunk girth.

Trunk girth was found to not be correlated to the elevation, but was negatively correlated to the slope (*r* = −0.22, *P* < 0.05). However, it was found to be better explained as the multivariate response of both slope and ER through a linear model (trunk girth = 16.843 − 0.049 × ER – 0.307 × Slope, *P* < 0.01**). Residuals of the model were examined and found normally distributed and homoscedastic. The interpolated map of residuals, displayed in Figure 7, still showed some spatial variation that was not explained by the predictors.

In comparison to the trunk girth variogram in Figure 5, the variogram of residuals (Figure 7), appears substantially changed as the latter is flattened, and the value of the sill is significantly smaller, showing no evidence of spatial clustering any more. This means that the spatial pattern of trunk girth data, observed in the field, seems mostly explained by the variability in the slope and ER. A further evidence of this was given when a correlation structure for the erratic component was added to the linear model. Different specification of the trend and spatial covariance model were tried, but this repeatedly led to the estimate of spatial correlation structures characterized by unrealistic ranges, well below the minimum distance between sampled vines, which was approximately 25 m.

Although much of the variability in trunk girth pattern was explained by ER and slope, a nonlinear spatial pattern was visible in the map of residuals (Figure 7). In order to improve the fit, a smooth function of the observation coordinates was added to the linear model. When a GAM model was estimated to account for the spatial trend of residuals, the linear terms were found to be significant and the estimated coefficients showed negative values. These results confirmed the inverse correlation between girth of trunk and both ER and slope.

Our results therefore suggest that the spatial pattern of trunk girth variability was almost completely reproduced by ER and slope alone, and that a nonlinear function of the field coordinates improved the model fit.

Similarly, the linear effects of ER and terrain attributes on the vine crop were tested and it was found to be best modeled as a linear function of ER and slope (Log (Yield) = 1.276*** − 0.008 ER** − 0.322 SLOPE*, *P* < 0.05). As in the case of trunk girth, the residual component of the vine yield fitting did not show any structured spatial variability (more flattened in comparison with the vine yield variogram in Figure 4(b)), except for a few observations that deviated from the relationship, which corresponded to vines for which the high yield was a spatially isolated phenomenon, in other words, neighbour plants did not exhibit similar values. As a result, the variables slope and ER adequately resolved the first and the second order spatial components of crop yield.

It has been shown that the effects of landscape position on crop yield strictly depended on year to year climate variability [31]. Significant correlations between landscape attributes and yield found in wet years may become insignificant or change their sign in dry years [32]. Fraisse *et al.* [33] showed how the number of uniform yield zones varied from year to year as a function of weather ranging from five areas in dry years to just two in a year with large water availability, and, not surprisingly, in dry years low productivity zones matched sloping shallow eroded areas. Relevant inference can therefore only be based on yield data from multiple years. Boydell and McBratey [34] developed yield zones from multi-year yield estimates and concluded that at least five years of yield data is required in order to identify stable patterns. On the other hand biometric attributes that integrate plant behavior over time, like trunk circumference, can be more suitable than yield to delineate areas that are spatio-temporally stable [35] and this would be most useful when multi-year data are unavailable.

We studied the correlation between resistivity and two plant attributes (trunk circumference and production) which are capable of reflecting the spatial variability of permanent edaphic properties more than any other plant parameter (e.g., characteristics linked to canopy vegetative expression). Perennial plants, such as vines, develop persistent spatial patterns in response to soil variability [7], and this is especially true for trunk girth, that integrates plant response to climatic and edaphic properties throughout the plant's life. Temporal stability of yield patterns has also been reported in the literature. Bramley *et al.* [36] evaluated vine production variability over a four year period, and showed that even if substantial year to year differences in mean annual yield were recorded, spatial yield patterns were temporally stable.

In this study the ER survey was carried out in November 2011, three years after the grapevine circumference and crop yield measurements were made. Nevertheless spatial patterns identified with ER were correlated with variability in vine attributes. This is due to the fact that spatial patterns of both ER and the chosen plant parameters are strongly dependent on permanent soil features (*i.e.*, soil texture, gravel lenses), whose position and contours exhibit little or no changes over time. Although soil moisture and temperature regime of course affect the absolute values of electrical resistivity and plant growth, they are not able to alter their spatial patterns if a large variability in soil permanent properties is found. The authors in [37] used 2D resistivity tomography in gravel exploration and reported that seasonal variation in soil water content caused a shift of gravel lenses resistivity from 1,500 to 300 Ohm·m: nevertheless the strong contrast between the high resistive rock fragments and the surrounding fine-textured material was stable enough to allow gravel lens to be identified at any time of the year. An increase of soil water content can even improve ER imaging ability of detecting resistive features, by increasing the contrast between resistive features and the background soil matrix [38]. This confirms the potential of electrical resistivity surveys to identify permanent soil features.

In the present work, ER proved to be a reliable predictor of within field spatial variability significantly affecting plant growth and yield. ER and slope explained almost completely the spatial variability of trunk girth. This was not surprising, since both variables are linked to soil and landscape attributes that dominate water availability in rainfed environments. As assessed by [7], vines growing on top and shoulder slopes tend to have shallower rooting systems and hence are more vulnerable to water stress. The use of ER as an explanatory variable of trunk girth improved predictions, compared to the use of slope solely, accounting for that part of variability which is linked to geopedology.

Soil electrical resistivity is affected by several soil properties, but at given sites often one factor is dominating, if its electrical response is stronger and its variation is large enough [39]. Resistive areas mapped in our field corresponded presumably to high rock fragment content within the profile. In a previous work [7] the authors dug soil profiles in the same area and observed in some trenches a high rock fragment content within the first meter. It is likely that these rock fragments in the soil were responsible for the high resistivity values measured in all of the explored layers, and at the same time affected vine growth and yield in those areas. Rock fragments are reported to increase soil resistivity in the order hundreds Ohm·m according to mineralogy and abundance [40]. The effect of stones is often strong enough to mask other soil components or even management-induced structural changes in agricultural soils [41]. Also, the magnitude of soil electrical signals may provide useful information as to the soil components that should be investigated in destructive sampling: electrical resistivity is affected by many soil materials, and their effects may compensate and mask each-other [39]; nevertheless, some values are usually only reached in the presence of selected materials. For example very high ER, as recorded in our case in selected areas at all depths, but especially in deep layers, indicates that resistive soil components (e.g., stones) should be seeked, and this provides a useful direction for the size of soil samples (large enough to quantify coarse fragments in a significant way) and the type of soil analysis.

Soil sampling using on-the-go DC sensors allow acquiring maps of ER at different depths without the limitations of EMI sensors in vineyards, related to the interference of metallic wires. This increases the possibility of exploiting the full potential of electrical soil mapping in viticulture, by filling a gap in spatial information on soil variability. Maps of ER are an invaluable tool for the spatial optimization of soil sampling, which is often inefficient due to the lack of information on the underlying structure of variation [42]. Continuous and extensive data on the spatial variability of soil electrical behaviour provides the basis for efficient sampling strategies such as stratified or surface-response sampling [43] within a time-frame compatible with the planning of sampling schemes in the field.

Relationships of ER or its reciprocal electrical conductivity of the soil with vine attributes relevant for management have been studied by other authors. Morari *et al.* [21] used a combination of EMI readings and static resistivity measurements to define uniform management units based on soil gravel content in a vineyard. Trought *et al.* [44] used continuous soil conductivity measurements to investigate the impact of soil texture on vine vigour, vine phenology and fruit composition. They found that conductivity, which was highly correlated to gravel content, was an effective predictor of trunk girth and grape quality spatial distribution. Acevedo *et al.* [35] used ER inter-row static measurements to determine the role of soil variability between zones. They found that differences in ER were correlated to different soil types and matched large variation in plant vegetative growth.

**Figure 8.**
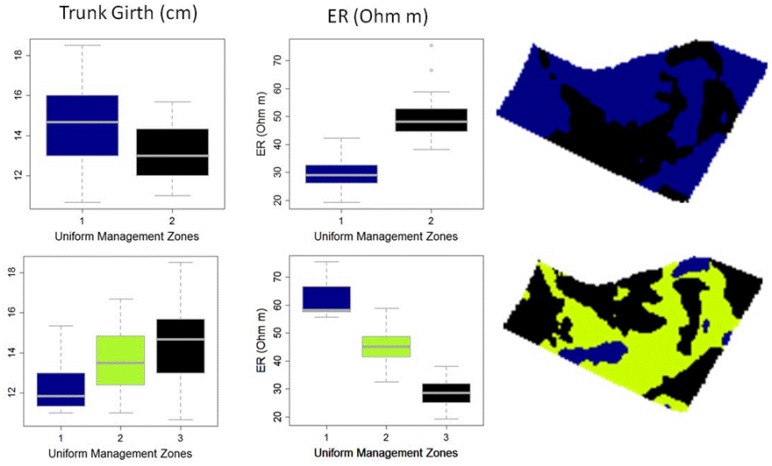
Boxplots of trunk girth and ER with increasing number of clusters (**left**) and uniform management zones maps derived by the clustering analysis (**right**). Top: management zone 1: blue; management zone 2: black. Bottom: management zone 1: blue; management zone 2: green; management zone 3: blue.

In order to choose the optimal number of clusters, suitable to define uniform management zones, we followed the approach suggested in [45]. All geo-referenced plant data were assigned to the respective management zone and differences in trunk girth and ER between uniform zones were analyzed by One-way analysis of variance (Figure 8).

When the vineyard under study was divided into two management zones, trunk girth average values significantly differed between zones (*t*-test *P* < 0.05), with the thinnest vines measured in the areas of high resistivity.

However, when three zones were delineated, no significant differences were found between trunk girth average values. Nevertheless a positive trend in trunk girth from zone I to zone III corresponded to an opposite trend in resistivity average values. Undoubtedly, other factors than edaphic properties might also influence vine yield and vegetative growth, such as pests and weather pattern during the growing season. Meanwhile, the use of easily measured field attributes remains a first step towards the identification of yield management zones for vineyards where multi-year spatial data are unavailable [30]. Moreover, identifying soil units that are spatio-temporally stable can serve as soil input for crop models, where alternative management strategies can be compared under different weather scenarios [46].

## 4. Conclusions

Soil electrical resistivity and slope allowed explaining almost completely the spatial variability of vine girth and yield, which are relevant parameters in viticulture. Both slope and soil electrical resistivity can be simultaneously measured with a single pass of the on-the-go DC soil sensor.

The definition of management zones is best based on multiple observations and criteria, from edaphic to logistic and economic, related to available farm technology and price of products and inputs. Nevertheless, first results from our study indicate that georeferenced information on ER and terrain morphology generated by the system can be the basis for the fast and non-destructive identification of differential vineyard management zones based on soil behaviour, which can be very useful in precision viticulture.
